# A One Health review of aerodigestive disease in dogs

**DOI:** 10.1111/jvim.16661

**Published:** 2023-03-28

**Authors:** Megan Grobman, Carol Reinero

**Affiliations:** ^1^ Department of Clinical Sciences Auburn University College of Veterinary Medicine Auburn Alabama USA; ^2^ Department of Veterinary Medicine & Surgery University of Missouri Veterinary Health Center Columbia Missouri USA

**Keywords:** dysphagia, radiographs, respiratory, videofluoroscopic swallow study

## Abstract

This review article seeks to define and describe aerodigestive disease in dogs, and review current and emerging methods of diagnostic evaluation. Aspiration of gastric contents into the respiratory tract is associated with the development and progression of numerous respiratory diseases in humans. In veterinary medicine the term “aspiration” is considered synonymous with “aspiration pneumonia” which, while frequently encountered, does not accurately reflect the breadth of aspiration associated respiratory syndromes (AARS). In the clinical veterinary literature, the effect of alimentary dysfunction on respiratory disease and vice versa (aerodigestive disease) is rarely investigated despite evidence in the human literature, animal models, and some studies and case reports linking alimentary and respiratory disease in small animals. Current methods of investigating aerodigestive diseases in veterinary patients are limited by inadeqate  sensitivity or specificity, potential for bias, cost, and availability. This necessitates investigations into advanced diagnostics to identify potentially underrecognized animals with AARS. Additionally, similarities in anatomy, physiology, and several disorders between dogs and humans, make experimental and naturally occurring canine models of AARS integral to translational research. Thus, evaluating dogs with aerodigestive disease might represent an area of substantial clinical relevance in human as well as veterinary medicine.

**Abbreviations**
AARS: aspiration associated respiratory syndromes
AeroD: aerodigestive disease
BOAS: brachycephalic obstructive airway syndrome
CB: chronic bronchitis
CN: cranial nerve
COPD: chronic obstructive pulmonary disease
CT: computed tomography
EER: extraesophageal reflux
EERD: extraesophageal reflux disease
EGJ: esophageal gastric junction
EOR: esophago‐oropharyngeal reflux
EORD: esophago‐oropharyngeal reflux disease
GER: gastroesophageal reflux
GERD: gastroesophageal reflux disease
GI: gastrointestinal
GOLPP: geriatric onset laryngeal paralysis polyneuropathy
HRM: high resolution manometry
ILD: interstitial lung disease
IPF: idiopathic pulmonary fibrosis
LES: lower esophageal sphincter
LES‐AS: lower esophageal sphincter achalasia‐like syndrome
LPR: laryngopharyngeal reflux
ME: megaesophagus
NPR: nasopharyngeal reflux
NPRD: nasopharyngeal reflux disease
OSA: obstructive sleep apnea
P‐A: penetration aspiration
PCR: pharyngeal constriction ratio
PPIs: proton pump inhibitors
RPS: reflexive pharyngeal swallow
sHH: sliding hiatal hernia
UES: upper esophageal sphincter
VFSS: videofluoroscopic swallow study

## 1 WHAT IS AN AERODIGESTIVE DISORDER?

Aerodigestive disorders (AeroD) represent a wide range of conditions wherein lesions develop between anatomic regions shared for breathing and swallowing. The aerodigestive tract encompasses respiratory and upper digestive structures including the pharynx (nasopharynx, oropharynx, and laryngopharynx), larynx, bronchi/bronchioles, pulmonary parenchyma, esophagus, and esophageal gastric junction (EGJ).^1^, ^2^ Aerodigestive disorders in people contribute to the pathogenesis and progression of respiratory conditions including chronic cough, asthma, bronchiolitis, idiopathic pulmonary fibrosis (IPF), and chronic obstructive pulmonary disease (COPD).^3^, ^4^, ^5^, ^6^, ^7^ Uncontrolled AeroD including gastroesophageal reflux disease (GERD), and its extraesophageal manifestation, extraesophageal reflux disease (EERD), contribute to diminished lung function, morbidity, and increased treatment costs.^8^ While treatment of AeroD improves prognosis in people, obvious clinical signs of alimentary disease might be absent, making identification diagnostically challenging.^2^, ^3^, ^9^, ^10^

Though historically focused on aspiration pneumonia, AeroD in dogs encompasses a wide variety of disorders (Table 1) which will be further discussed in this review.^2^, ^14^, ^23^, ^76^, ^77^, ^78^, ^79^ As in people, in some cases treatment for alimentary diseases improves prognosis, for example, in dogs undergoing surgical correction of brachycephalic obstructive airway syndrome (BOAS).^80^ Additionally, the challenges in diagnosing AeroD in people are mirrored in dogs presenting without obvious alimentary clinical signs (ie, occult disease).^2^ Increasing clinical awareness of AeroD in dogs and a multimodal clinical approach are necessary for identification and optimal treatment.

**TABLE 1:** Aerodigestive diseases in dogs.

**FIGURE 1:**
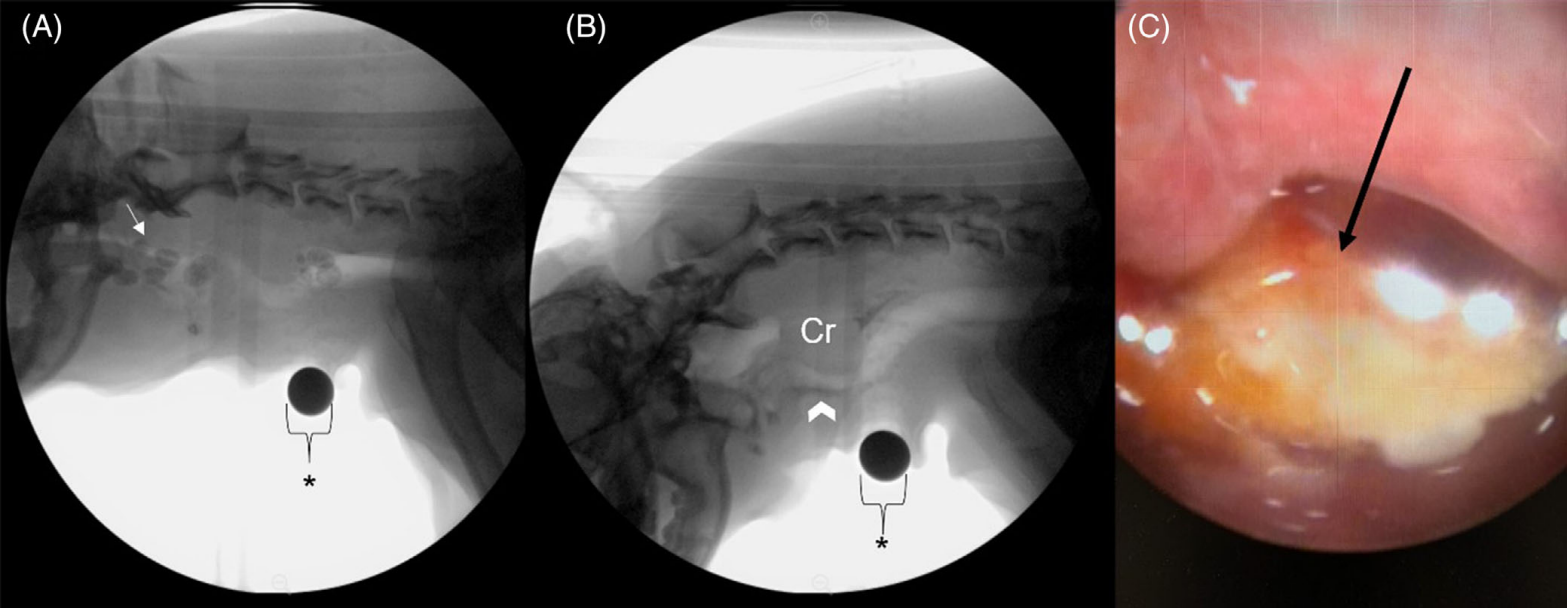
(A‐C) A still VFSS image from a 13‐year‐old MC Boston Terrier diagnosed with cricopharyngeal achalasia after being evaluated for chronic cough and mucopurulent nasal discharge. (A) The dog is freely consuming a kibble consistency. Kibble is seen above the soft palate following pharyngeal swallow demonstrating nasopharyngeal reflux (white arrow). (B) The dog is freely consuming a liquid consistency. A hypertrophied cricopharyngeus muscle (Cr) demonstrates a classic “thumbprint” pattern. Macroaspiration is observed with material entering the cervical trachea (arrowhead). (C) An image from a retroflexed choanal examination of the nasopharynx identified a foreign body (carrot; black arrow). A 1 cm size marker is denoted by the asterisk (*). The image from the choanal examination was provided by Dr. Vientos‐Plotts, University of Missouri.

Upper airway disorders (pharynx and larynx)
Brachycephalic obstructive airway syndrome (BOAS)^11^, ^12^, ^13^
Nasal/nasopharyngeal disease
Rhinitis^14^
Nasal masses^15^
Nasopharyngeal stenosis^16^
Nasopharyngeal foreign bodies^16^ (Figure 1)
Laryngeal disease
Laryngeal paresis and paralysis^17^, ^18^
Laryngeal collapse (nonbrachycephalic)^15^, ^18^, ^19^
Pharyngeal collapse^2^
Sleep disordered breathing^20^, ^21^, ^22^
Lower airways and pulmonary parenchyma
Large airway obstruction^23^
Tracheitis^2^, ^23^
Chronic bronchitis^2^, ^23^, ^24^
Bronchiectasis^23^, ^25^
Diffuse aspiration bronchiolitis^23^, ^26^
Aspiration pneumonia/pneumonitis^27^
Interstitial lung disease^24^
Idiopathic cough^28^, ^29^
Upper digestive tract and esophageal gastric junction (EGJ)
Pharyngeal weakness (see Table 2)
Pharyngeal masses^46^, ^47^
Cricopharyngeal achalasia and dyssynchrony^44^, ^48^
Esophageal dysmotility^49^
Megaesophagus (see Table 3)
Esophagitis^74^
Lower esophageal sphincter achalasia‐like syndrome (LES‐AS)^52^, ^75^
Reflux diseases
Gastroesophageal reflux disease (GERD)^2^, ^15^
Extraesophageal reflux disease (EERD)^2^, ^15^
Nasopharyngeal reflux disease (NPRD)^2^, ^15^
Esophago‐pharyngeal reflux disease (EORD)^2^
Sliding hiatal hernia (sHH)^15^
Structural disorders of the esophagus
Strictures^50^
Persistent right aortic arch^50^
Esophageal diverticula^52^

## 2 MECHANISMS OF AIRWAY PROTECTION

**FIGURE 2:**
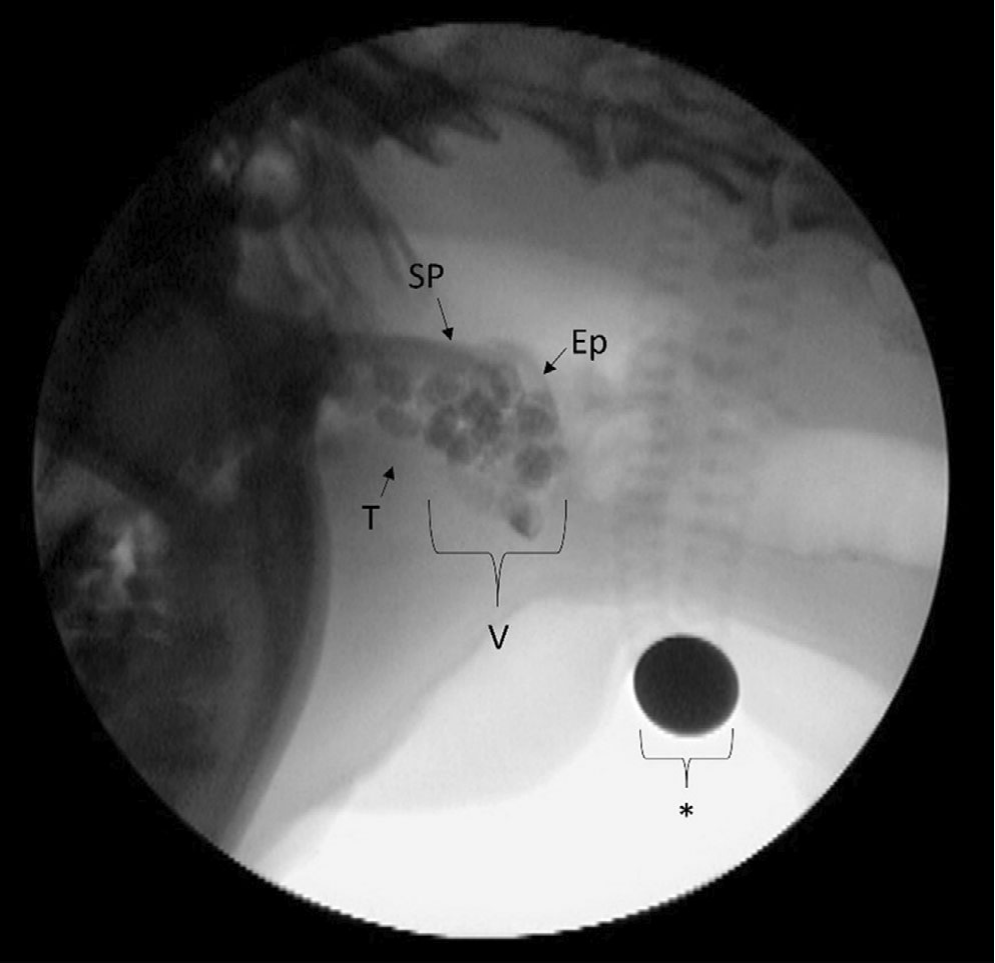
A still image from a videofluoroscopic swallow study of a dog showing accumulation of kibble in the valleculae (V). The valleculae is bordered caudally by the epiglottis (Ep), dorsally by the soft palate (SP) and rostrally by the base of the tongue (T). A 1 cm size marker is denoted by an asterisk (*).

Aerodigestive diseases often represent failures of airway protection. Airway protective mechanisms include normal swallowing (Figure 2), respiratory/swallowing coordination or “gatekeeping,” and basal and response mechanisms (Table 4).

### 2.1 Normal swallowing

Normal swallowing is an important means of airway protection, with dysphagia being an important risk factor for aspiration‐associated respiratory syndromes (AARS).^81^, ^114^ A swallow is classically divided into 3 sequential phases.^87^, ^115^ The oral/preparatory phase is largely voluntary and responsible for the accumulation of a formed food bolus within the vallecullae which is bordered by the soft palate, elevated epiglottis and base of the tongue (Figure 2).^82^, ^87^ The pharyngeal swallow phase might occur spontaneously (reflexive swallow) or be associated with feeding behavior.^81^, ^96^ A pharyngeal swallow triggers the esophageal phase by initiating primary esophageal peristalsis. Esophageal peristalsis, either primary or secondary, conducts the bolus into the stomach through an open lower esophageal sphincter (LES) followed by LES closure with transient hypertonicity to prevent reflux.^87^

### 2.2 Respiratory swallow coordination “gate keeping”

“Gatekeeping” refers to the centrally mediated partnership that ensures that respiration and swallowing do not occur simultaneously.^83^ Alterations in breath‐swallow coordination are documented in people with dysphagia and chronic respiratory disease linking disordered swallow and respiratory coordination with increased risk of AARS.^82^, ^83^, ^116^, ^117^

An additional means of breath‐swallow coordination is swallow‐apnea.^82^ Multiple swallows in series prolongs swallow‐apnea.^118^ This places increased stress on patients with respiratory disease and is associated with disordered coordination of respiration and swallow.^83^, ^116^ This might increase risk of aspiration in patients with existing respiratory disease contributing to disease exacerbations.^116^

### 2.3 Basal and response mechanisms

Basal and response mechanisms are barriers to aspiration which are present independent of and dependent on chemical and mechanical stimulation, respectively. Examples of basal and response mechanisms include the upper and lower esophageal sphincters (UES and LES), and primary and secondary peristalsis, respectively.^83^, ^106^ A summary of airway protective mechanisms including the basal and response mechanisms are provided in Table 4.

## 3 CLINICAL APPROACH

### 3.1 Signalment

Aerodigestive diseases can occur in dogs of any age and breed. However, brachycephalic dogs might be considered predisposed, develop AeroD at a younger age, or might have more severe clinical signs related to AeroD compared to nonbrachycephalic dogs.^2^, ^15^

### 3.2 History

The clinical presentation of dogs with AeroD is variable. Dogs might present with exclusively clinical signs of respiratory disease, exclusively alimentary disease, or a combination.^2^, ^15^, ^119^ Further, the range of conditions is diverse with many presentations and clinical evaluations being disease‐specific making clinical suspicion of AeroD important. Dogs with a history of recurrent aspiration pneumonia, coughing while eating and drinking, dysphagia, recent anesthesia, vomiting and regurgitation, or clinical signs suggestive of reflux disease (eg, repetitive dry swallowing, lip licking, gagging, nighttime restlessness, etc) should be considered at high risk for AeroD.^2^

### 3.3 Physical examination

Physical examination findings might depend on number of factors and are often dog specific. Dogs with physical examination findings that might contribute to dysphagia (eg, cranial nerve deficits and severe periodontal disease), evidence of laryngeal dysfunction (eg, stridor and dysphonia), evidence of polyneuropathy (eg, conscious proprioceptive deficits), myopathy (eg, masticatory or generalized muscle atrophy), or evidence of upper airway obstruction (eg, brachycephalic obstructive airway syndrome, laryngeal paralysis, laryngeal or nasal masses) could be considered at increased risk for AeroD.^2^, ^15^, ^23^, ^41^, ^120^

## 4 DIAGNOSTIC EVALUATION

### 4.1 Thoracic radiographs

Thoracic radiographs, often the first line diagnostic for dogs with respiratory disease, are poorly sensitive for AeroD where signs are elicited by mechanical/chemical stimulation of the pharynx and larynx.^2^ Additionally, the static nature of radiographs limit the ability of the modality to detect and evaluate functional and dynamic processes inclusive of dysphagia, reflux, and repetitive microaspiration.^2^, ^15^, ^121^, ^122^, ^123^, ^124^, ^125^ This is supported by recent studies in dogs with respiratory clinical signs with AeroD documented using videofluoroscopic swallow studies (VFSS) that had normal thoracic radiographs.^2^, ^15^, ^119^ Laryngeal and pharyngeal stimulation, reflux, and occult aspiration triggering cough might be missed by thoracic radiography.^14^, ^126^ Further, thoracic radiographs might miss or underestimate the severity of pulmonary pathology. Computed tomography (CT) provides superior resolution and lesion localization compared to thoracic radiographs for many AeroD.^23^, ^127^

### 4.2 Treatment trials, pH monitoring, and endoscopy for GERD and EERD

Adjunctive diagnostic and monitoring strategies for GERD and EERD in veterinary medicine incorporate client surveys, treatment trials with proton pump inhibitors (PPIs), esophagoscopy, and rarely esophageal manometry and ambulatory pH probes.^128^, ^129^, ^130^, ^131^ However, these have important limitations. Though a mainstay in clinical practice, treatment trials with client reporting are inherently prone to bias because of variable client vigilance, and a failure to recognize episodic or subtle clinical signs. Treatment trials with PPIs might be associated with a large and variable placebo effect. In people, randomized control trials including a placebo arm have documented improvement in 1% to 40% in the placebo group, which is often statistically significant.^132^, ^133^ Further, treatment trials might take up to 8 weeks to detect an effect.^23^, ^130^ Diagnostic tests relying upon esophageal or pharyngeal pH (eg, ambulatory pH monitoring) fail to recognize reflux in human patients treated with PPIs or those with nonacidic reflux, the latter of which is being increasingly implicated, representing up to 90% cases in some human studies.^9^, ^134^, ^135^, ^136^ People with GERD and symptoms of heartburn have a prevalence of nonacidic reflux of 37.4% and 44.3% in patients with erosive esophagitis and nonerosive esophagitis, respectively.^137^ Similar studies discriminating between nonacidic and acidic reflux and erosive and nonerosive esophagitis are lacking in dogs.^131^ However, a study evaluating administration of esomeprazole with or without cisapride on reflux events in anesthetized dogs, found no change in the number of reflux events compared to placebo when esomeprazole was given as a sole agent despite significant increases in pH.^138^ This study and others demonstrate active reflux in dogs despite treatment with PPIs.^138^, ^139^ Canine gastric fluid contains both bile acids and digestive enzymes that have the potential to cause damage when the pH is relatively neutral.^28^, ^140^, ^141^ Though further studies are needed, nonacidic reflux is reported in dogs and has the potential to contribute disease.^138^, ^141^, ^142^, ^143^

Diagnostics requiring anesthesia impart increased risk of aspiration events because of decreased airway protective mechanisms. In human studies endoscopy identified abnormalities in less than 50% of patients with known disease (eg, nonerosive reflux disease).^144^ Studies utilizing high resolution manometry (HRM) and pH probes have made significant contributions to our understanding of pharyngeal and esophageal motility and GERD. HRM is feasible in both awake or sedated dogs, with esophageal pressure profiles being successfully collected for both liquid and solid food boluses.^145^ HRM studies also demonstrated increased LES resting pressures after oral administration of cisapride but not metoclopramide compared to placebo.^146^ Ambulatory pH monitors are utilized to determine response to acid suppression and to evaluate the association between GER events and respiratory clinical signs in healthy dogs.^128^, ^143^, ^147^ There is a poor association between GER and respiratory clinical signs. However, as noted above, this method detects only acidic reflux and cannot distinguish between physiologic and pathologic reflux events. Additional multimodal studies are warranted to further investigate the relationship between GER and clinical signs of respiratory disease. However, despite the contributions of these modalities in a research setting, their broad use in dogs is limited by availability, cost, and need for substantial operator training.^128^, ^145^

### 4.3 Videofluoroscopic swallow studies

Observation of feeding behavior, particularly by VFSS, is critical to localizing disease in dogs with dysphagia.^120^ Videofluoroscopic swallow studies are the criterion standard for evaluation of dysphagia in dogs.^148^ Historically, these have been performed with dogs held in lateral recumbency and force‐feeding, limiting use because of unacceptable risks of aspiration. A recent alternative which, by allowing unrestrained free‐feeding, reduces risk of aspiration to what would be expected from feeding at home.^148^ Natural feeding position, physiologic bolus sizes, and standardized food items with rheological properties (ie, material properties reflecting flow and deformation; eg, viscosity) objectively consistent with commercially available products, further increases the physiologic relevance of VFSS in dogs.^148^ Objective metrics in VFSS help identify dogs with pharyngeal weakness, cricopharyngeal achalasia and dyssynchrony, esophageal weakness, reflux, and lower esophageal sphincter achalasia‐like syndrome (LES‐AS).^2^, ^52^, ^148^

In people, VFSS using an objective 8‐point scale of laryngeal penetration and aspiration (P‐A) has been used to evaluate airway protection. A score of ≥3 is considered abnormal and is predictive of ARRS in children and adults.^149^, ^150^, ^151^, ^152^ A 7‐point scoring system is validated in animals (Table 5).^153^ The application of this system helped identify an incidence of P‐A of 39% in dogs undergoing VFSS as well as risk factors for P‐A including pharyngeal weakness.^119^

### 4.4 Scintigraphy

Nuclear scintigraphy, which has been employed to measure mucociliary clearance in dogs,^154^ is also capable of measuring reflux and cumulative small volume aspiration (ie, microaspiration).^10^, ^155^, ^156^, ^157^ It does not rely on pH, is noninvasive, and can be performed without anesthesia.^155^, ^156^, ^157^, ^158^, ^159^ This technique is safe, having been used to evaluate pulmonary aspiration in fragile human infants.^155^, ^158^ In people, cough and laryngospasm were strongly correlated with positive reflux/aspiration on scintigraphy.^158^ Further, patients who were positive for reflux on scintigraphy had symptomatic response to surgical treatment.^158^ A study has validated this technique in clinically normal nonbrachycephalic dogs where reflux events were documented after eating a meal containing colloidal ^99^m‐technetium phytate.^10^ Reflux was diagnosed by increased esophageal counts (≥200% of background) with concurrent decreases in gastric counts. Though limited in availability to tertiary referral hospitals, this provides a potential alternative to evaluating dogs for reflux, aspiration, or both.

### 4.5 Biomarkers of reflux

Biomarkers are used to diagnose, prognosticate, and identify groups at risk for disease and have had a beneficial impact in companion animal medicine when used appropriately.^160^ Biomarkers might advance our understanding of disease pathogenesis and allow objective investigation of efficacy of novel therapeutics in various diseases. Biomarkers have been used reliably to provide evidence of reflux and aspiration in people.^134^, ^161^, ^162^ In dogs proteomic profiles differed between gastric fluid and the oropharynx, and the oropharyngeal proteome differed between healthy dogs, dogs with cough, and dogs with vomiting/regurgitation.^28^ Dogs presenting exclusively for cough had detectable gastrointestinal proteins on oropharyngeal swabs, a finding that was not present in healthy dogs. The presence of gastrointestinal specific proteins in dogs with cough suggests EERD with or without aspiration as a contributor to respiratory signs.^28^, ^163^

### 4.6 Acoustic cough monitoring

Cough represents both a critical airway protective mechanism for the respiratory tract as well as a marker for disease control. Unfortunately, management of cough in veterinary medicine is often suboptimal and based on subjective response to therapy.^164^, ^165^ Acoustic wave analysis incorporating spectral and waveform evaluation has provided an objective means of detecting cough in people and has been recently adapted for veterinary use.^165^, ^166^, ^167^, ^168^, ^169^, ^170^, ^171^ This might provide a means of objectively evaluating cough frequency in veterinary patients and bridge the gap between subjective monitoring and objective assessment.

## 5 RECOGNIZED AERODIGESTIVE DISORDERS IN DOGS

### 5.1 Upper airway disorders

#### 5.1.1 Brachycephalic obstructive airway syndrome

Brachycephalic obstructive airway syndrome (BOAS) is a cause of impaired respiration in brachycephalic breeds primarily because of upper airway abnormalities.^11^ There is increasing recognition of comorbid esophageal, gastric, and intestinal diseases which can exacerbate or be exacerbated by the primary respiratory disease and are seen in up to 97% of cases.^172^ Specific abnormalities associated with BOAS are listed in Table 1. Conformational changes created by selective breeding leads to increased respiratory resistance and chronic induction of negative airway pressures.^173^ The latter causes soft tissues to be further drawn into the airway lumen, exacerbating upper airway collapse.^173^ Negative intrathoracic pressures are postulated to be associated with GER, esophagitis, and hiatal hernia.^174^, ^175^ Importantly, surgical correction of upper airway abnormalities can lead to resolution or substantial improvement in digestive signs.^12^, ^176^

Clinical signs of BOAS include stertor, stridor, exercise intolerance, gag or cough, labored inspiratory efforts, respiratory distress, obstructive sleep apnea, cyanosis, hyperthermia, exercise intolerance, and syncope.^11^, ^177^, ^178^ Ptyalism, dysphagia, regurgitation and vomiting are also common.^11^ Diagnosis relies on signalment, physical examination, and diagnostic imaging (cervical and thoracic radiography, respiratory fluoroscopy, VFSS, CT of the head and neck, functional upper airway examination, retroflex rhinoscopy, tracheobronchoscopy, and esophagoscopy/gastroscopy/duodenoscopy). Specialized maneuvers (ie, temporary endotracheal tube obstruction, manual pressure on cranial abdomen, and Trendelenburg position [body angled at 30° while in left lateral recumbency]) under general anesthesia during endoscopic examination might be needed to highlight EGJ abnormalities and sliding hiatal hernia.^179^ Gastric and duodenal biopsies might be required to document a chronic enteropathy.

#### 5.1.2 Nasal/nasopharyngeal disease

In dogs, EER might contribute to inflammatory and obstructive nasal and nasopharyngeal disorders, such as chronic rhinitis, nasopharyngeal foreign bodies, and nasopharyngeal stenosis, because of contents of refluxate including acid, digestive enzymes, and foreign material.^14^, ^16^ The link between nasal/nasopharyngeal disease and EER is underrecognized in dogs.^14^ Risk of chronic rhinosinusitis is increased in people with GER.^180^ Speculated mechanisms include a direct cytotoxic effect of refluxate contacting the nasal passages or an indirect mechanism of an autonomic nervous system reflex.^181^, ^182^

Clinical signs might include sneezing, reverse sneezing, nasal discharge, stertor, labored inspiratory efforts and gagging. Diagnostic evaluation includes physical examination, VFSS, imaging of the head (radiography, fluoroscopy, or CT) and endoscopy (anterograde and retrograde rhinoscopy).

#### 5.1.3 Laryngeal disease (paralysis, paresis, and collapse)

Laryngeal paralysis is a congenital or acquired disorder wherein the arytenoid cartilages fail to abduct on inspiration leading to an upper airway obstruction.^19^ Laryngeal paresis is associated with weak but present abduction of the arytenoid cartilages and was previously thought to occur as the early stage of a progressive degenerative disease leading to laryngeal paralysis in older, large breed dogs.^183^ Laryngeal dysfunction, including laryngeal paresis and laryngeal spasm might occur secondary to EER.^17^, ^184^ Laryngeal collapse is not a primary disorder but occurs secondary to chronic upper airway obstruction (eg, BOAS) that increases negative intraglottic luminal pressures. There are 3 stages: (1) everted laryngeal saccules, (2) aryepiglottic collapse with medial deviation of cuneiform processes, and (3) collapse of corniculate processes.^19^

Clinical signs of laryngeal disease depend on the degree of airway obstruction and typically include stridor, inspiratory respiratory distress, exercise intolerance, cyanosis, and collapse.^19^ A voice change is frequently observed.^19^ Clinical signs of respiratory disease might be exacerbated by heat or exercise/excitement. Abnormal functioning of pharyngeal and esophageal muscles might lead to increased risk of micro‐ or macroaspiration and could lead to gag and cough.^19^, ^185^ Diagnosis relies on signalment, physical examination, neurologic examination, CBC/biochemical profile/urinalysis/thyroid testing, cervical and thoracic radiography, and upper airway examination.^19^

#### 5.1.4 Sleep‐related breathing disorders

Sleep‐related breathing disorders are associated with episodic hypopnea or apnea during sleep, and in dogs, are generally because of collapse of upper airways (ie, obstructive sleep apnea [OSA] in dogs with BOAS).^20^, ^21^ Arousal restores normal airflow.^186^ During apneic episodes, increases in airway pressure as breathing efforts are intensified in response to an obstructed airway might lead to GER.^186^ Altered swallowing function has been reported in people with OSA.^187^ Additionally, depressed consciousness is associated with an unprotected airway, increasing the risk of aspiration.^117^ In people with OSA, there is a high prevalence of chronic occult aspiration, but parallel reports in dogs are lacking, with studies assessing risk factors for aspiration pneumonia failing to identify OSA as an etiology.^188^, ^189^, ^190^, ^191^ Diagnosis relies on compatible clinical signs (>5 episodes of hypopnea/apnea during sleep unexplained by another condition) and at least 1 of the following observed by owners: (1) waking up with gasping or choking, (2) low activity or sleeping while sitting/standing, (3) apneic episodes or breathing interruptions, or (4) seeking a specific sleeping position to aid in breathing efforts.^192^ Other diagnostics to assess the cause of upper airway obstruction might require sedation/anesthesia to induce the event and include radiography, fluoroscopy, CT, and functional upper airway examination. Obstructive sleep apnea might lead to clinically important pulmonary hypertension suggesting a role for echocardiography.^193^

### 5.2 Lower airway disorders

#### 5.2.1 Airway foreign bodies

Large airway obstruction from airway foreign body aspiration might present as respiratory emergencies because of obstruction of airflow. Size of the dog, size and location of the foreign body, and any dynamic repositioning will dictate severity of clinical signs.^23^, ^194^ More commonly, airway foreign bodies move to smaller airways and might migrate into the pulmonary parenchyma or pleural cavity.^195^ Large airway obstruction manifests with acute respiratory distress as the predominating clinical sign, whereas movement of foreign bodies to lobar/segmental/subsegmental airways often presents with chronic cough, possibly with systemic signs of illness reflecting a secondary bacterial pneumonia. History such as witnessed aspiration, survey radiography, fluoroscopy, CT and tracheobronchoscopy are helpful diagnostics.

#### 5.2.2 Tracheitis

Among other causes, noninfectious inflammation of the trachea can occur with repetitive micro‐ or macroaspiration.^2^, ^23^ Classically, with aspiration, erythema and increased mucosal vascularity are most prominent proximally, gradually dissipating distally; this is reflective of the depth of penetration of refluxate.^2^ Experimentally, gastric juice impairs tracheal mucociliary function in the dog.^196^ Cough is the most common clinical sign. Direct visualization of the tracheal mucosa with tracheoscopy and documentation of GER, EER, or aspiration on VFSS are supportive diagnostics.

#### 5.2.3 Chronic bronchitis

Chronic bronchitis (CB) is defined as a clinical syndrome of chronic cough greater than 2 months in duration associated with neutrophilic airway inflammation and mucus hypersecretion.^197^ Repetitive microaspiration has been associated with canine CB.^2^, ^23^, ^24^ Additionally, respiratory dysbiosis (perturbations of the airway microbial communities) might in part be caused by microaspiration, with additional research needed to elucidate the role of microbes in triggering, exacerbating or perpetuating airway injury.^198^ Diagnosis relies on history, physical examination, thoracic radiography, bronchoscopy, and airway lavage for cytology and culture.

#### 5.2.4 Bronchiectasis

Bronchiectasis is defined as irreversible dilation of airway walls because of cyclical inflammation, infection, and impairment of mucociliary function.^25^, ^199^ Aspiration is recognized as an important cause.^2^, ^23^, ^25^ Cough nearly universally occurs, with tachypnea or labored respiration less frequently reported.^25^ Bronchiectasis might be missed on survey radiography, but CT is highly sensitive.^25^ Bronchoscopy can allow airway sampling, critical for documentation of inflammation and potentially infection. Dogs with bronchiectasis are at increased risk of bacterial infection.^200^

#### 5.2.5 Diffuse aspiration bronchiolitis

Small airway diseases, affecting bronchioles <2 mm in diameter and lacking cartilage in their walls, have been described and classified in people and cats,^26^, ^201^ but are not well characterized in dogs.^202^, ^203^, ^204^, ^205^, ^206^ Dogs have no pathognomonic respiratory clinical signs. There are many risk factors associated with small airway injury, with aspiration leading to a subtype of primary bronchiolar disease called diffuse aspiration bronchiolitis.^26^ Survey radiography and respiratory cytology are incapable of diagnosing bronchiolar disorders, making CT and histopathology key diagnostics in dogs and cats with a risk factor for aspiration and other testing failing to provide an explanation for clinical signs.^26^, ^201^ Histologically, diffuse aspiration bronchiolitis is characterized by inflammation and a foreign body reaction in bronchioles.^26^

### 5.3 Pulmonary parenchymal diseases

#### 5.3.1 Aspiration pneumonia/pneumonitis

Inhalation of orophargyneal or gastrointestinal contents causes chemical, bacteriologic, and immunologic injury of the pulmonary parenchyma falling under the umbrella term “aspiration pneumonia” in dogs.^191^ The type of inflammatory response following aspiration depends on the volume and composition of the aspirate, leading to aspiration pneumonia (with clinically impactful bacterial infection) or aspiration pneumonitis (chemical pneumonitis without clinically impactful bacterial infection).^27^, ^207^ In veterinary practice, the term aspiration pneumonia is used to encompass both syndromes as clinical signs and radiographic features are often overlapping.^27^ Clinical signs can be systemic (eg, fever, lethargy) or local (eg, cough, tachypnea, labored respiration). The primary clinically relevant distinction between these 2 subtypes is whether antimicrobials are indicated for therapy.^27^ While antimicrobial use is widespread in veterinary practice, there is increasing recognition that many cases of aspiration pneumonia in dogs lack clinically important infection.^27^, ^208^ In addition to identifying a predisposing factor for aspiration, clinical signs, radiographic features (dependent lung lobes with an interstitial or alveolar pattern), hematologic data (leukocytosis or leukopenia, band neutrophilia) and airway cytology and culture are useful for determining optimal treatment.

#### 5.3.2 Interstitial lung diseases

Interstitial lung diseases (ILDs) are a heterogenous group of diffuse parenchymal disorders associated with varying degrees of inflammation and fibrosis in absence of infection or neoplasia.^209^ There are a variety of triggers of pulmonary injury, including aspiration, and fibrosis represents a final common pathway for many ILDs. One subtype of fibrotic ILD in people called idiopathic pulmonary fibrosis (IPF) was suggested to be associated with GERD in a recent meta‐analysis.^210^ GERD is linked to progression of a variety of progressive fibrosing ILDs in people including IPF, hypersensitivity pneumonitis, connective tissue disease‐ILD, cryptogenic organizing pneumonia and others.^211^ In West Highland white terriers, a breed with familial pulmonary fibrosis, aspiration of gastroesophageal refluxate into the airways of affected dogs with silent repetitive microaspiration was documented by assessing for presence of bile acids in airway lavage.^24^ Another ILD definitively linked to aspiration in dogs is exogenous lipid pneumonia which has been caused by inadvertent aspiration of mineral oil.^212^ Clinical signs can include cough, tachypnea, rapid and shallow breathing, exercise intolerance, respiratory distress, and cyanosis. History, physical examination, radiography, CT, and airway cytology can rule out other disorders and make an ILD more likely; however, definitive diagnosis relies on histopathology.

### 5.4 Idiopathic cough

When a comprehensive diagnostic evaluation fails to identify an underlying structural, functional, infectious, inflammatory, fibrotic, or neoplastic disorder as an etiology of cough and when treatment of underlying causative factors is ineffective in controlling cough, it is termed “idiopathic” or unexplained cough.^213^ Idiopathic cough must be discriminated from cases in which a thorough diagnostic evaluation and treatment trials have not been performed resulting in a lack of explanation for the cause of cough.

It is unclear if idiopathic cough in dogs bears similarities to chronic cough hypersensitivity syndrome in people, in which the initial cough trigger has disappeared but the cough reflex is enhanced.^29^, ^214^ Alternatively, because of the low index of suspicion and widespread inability to test for EER, laryngopharyngeal reflux (LPR; backflow of gastric contents into the laryngopharnyx), and repetitive microaspiration in dogs, it is possible that a subset of idiopathic canine cough is because of effects of reflux.

### 5.5 Pharyngeal weakness

Pharyngeal weakness refers to impaired pharyngeal peristaltic activity in the absence of obstruction, improper timing of pharyngeal swallowing, or both.^215^ Studies in dysphagic dogs identified pharyngeal weakness in 13% of cases reviewed by VFSS.^216^ Pharyngeal weakness in dogs is the nonspecific consequence of neuromuscular diseases,^43^, ^217^ myopathies,^22^, ^41^ cranial nerve deficits,^35^ infectious agents,^31^, ^32^ and long term negative pressure gradients^22^, ^35^ (Table 5). Brachycephalic dog models of OSA demonstrate architectural remodeling of the pharyngeal musculature leading to pharyngeal weakness, +/− collapse, and exacerbations of OSA and dysphagia.^22^ Extraesophageal reflux disease in people is also thought to contribute to pharyngeal weakness through the development of a local myositis; however, comparable studies are missing in dogs.^218^

Regardless of etiology, pharyngeal weakness is considered an independent risk factor for aspiration in dogs and people.^2^, ^119^, ^150^, ^215^ Pharyngeal weakness is most frequently diagnosed by VFSS.^44^ An increased pharyngeal constriction ratio (PCR), the presence of residual contrast material in the pharynx following an appropriately timed pharyngeal swallow, or both are supportive of a diagnosis of pharyngeal weakness.^44^, ^119^ The timing of pharyngeal swallow is important to distinguish pharyngeal weakness from delayed opening of the upper esophageal sphincter (UES, that is, cricopharyngeal dyssynchrony).^44^ High resolution manometry provides detailed information regarding both the pressure and timing of pharyngeal swallow; however, it is not widely available in veterinary medicine.^49^

### 5.6 Pharyngeal masses

Pharyngeal masses inclusive of abscesses, cysts, polyps, neoplasia, and sialoceles might all present with clinical signs associated with pharyngeal dysphagia.^46^ A markedly elongated soft palate might also act as a pharyngeal “mass effect.” Ventral cervical masses in dogs have also been reported to cause pharyngeal dysphagia.^47^ These lesions disrupt normal pharyngeal swallow, increasing risk of impaired airway protection and AARS.^44^ Dysphagia after surgery or radiation therapy affecting the ventral cervical area or oral cavity is common in humans.^219^ A study in dogs undergoing definitive radiation therapy for thyroid carcinoma reported dysphagia as acute radiation side effect suggesting similar outcomes in dogs.^220^ Videofluoroscopic swallow studies are the initial diagnostic of choice to assess dogs with suspected pharyngeal dysphagia.^44^ Sedated or anesthetized oral examinations with or without sample collection are ultimately required for a diagnosis in most cases. Endoscopy, CT or both might also be necessary to evaluate extraluminal compressive masses, the extent of intraluminal disease, as well as metastasis.

### 5.7 Cricopharyngeal achalasia and dyssynchrony

The cricopharyngeus muscle is the major muscular component of the UES. Absent or incomplete, or inappropriately timed opening (delayed opening or early closure) of the cricopharyngeus muscle reflect cricopharyngeal achalasia and dyssynchrony, respectively. A study in dysphagic dogs identified cricopharyngeal achalasia, dyssynchrony, or both, in 8% (n = 17) of evaluated dogs: 6 with asynchrony, 6 with achalasia, and 5 with both asynchrony and achalasia.^216^ Both conditions result in impaired bolus clearance during pharyngeal swallow and high risk of aspiration of pharyngeal contents into the airways (Supplementary Video 1).^120^, ^221^ The underlying etiology in dogs is suspected to be a congenital focal neuropathy; however, acquired cases in older dogs have been reported including secondary to hypothyroidism.^48^, ^222^ In people, there is an association between acquired cricopharyngeal dyssynchrony and reflux disease (GERD and EERD). These studies documented abnormalities in the timing of cricopharyngeal opening, with as many as 50% of dogs with GERD showing delayed opening or early closure of the cricopharyngeus muscle.^223^, ^224^ As for pharyngeal weakness, detection of cricopharyngeal achalasia and dyssynchrony is best achieved by HRM or VFSS with special attention paid to the PCR, timing of pharyngeal swallow, and the maximal diameter of the UES during bolus passage.^129^, ^216^, ^225^

### 5.8 Esophageal dysmotility

In people, ineffective esophageal peristalsis represents a heterogenous population of motility disorders often diagnosed by HRM.^226^, ^227^ In dogs, esophageal dysmotility is less well defined but generally refers to impaired esophageal motility resulting in accumulated swallowed boluses within the esophagus and or a prolonged esophageal transit time on VFSS.^2^ Importantly, esophageal dysmotility might occur in the absence of overt megaesophagus.^2^, ^52^ Etiologies are suspected to be similar to those for megaesophagus and might actually precede esophageal dilation in these cases (Table 3).^228^ In addition, esophageal dysmotility has been documented with esophagitis, geriatric onset laryngeal paralysis polyneuropathy (GOLPP), sliding hiatal hernia (sHH), esophageal diverticula, esophageal fistulas, and LES‐AS.^13^, ^15^, ^52^, ^58^, ^71^, ^216^, ^229^ Some breeds appear predisposed to esophageal dysmotility including the Bouvier and Chinese Shar Pei.^40^, ^230^ Several studies have also documented esophageal dysmotility alone or in combination with GERD and sHH in brachycephalic dogs of multiple breeds.^15^, ^231^ Mild esophageal dysmotility attributable to maturation of the neuromuscular system might be detected in juvenile dogs that might improve up to 1 year of age.^229^

Defects in motility and esophageal clearance represent failures in airway protection. Esophago‐oropharyngeal reflux (EOR) is associated with increased risk of pathologic laryngeal penetration and aspiration in dogs undergoing VFSS.^119^ Concurrent laryngeal dysfunction compounds these risks.^58^ In reflux esophagitis, a failure to effectively clear the refluxate from the esophagus might contribute to disease progression and the development of structural abnormalities such as esophageal strictures.^71^ A VFSS is considered the diagnosis of choice in dogs to detect subtle and segmental abnormalities in esophageal motility.^216^ Detailed information on esophageal motility might also be achieved by HRM; however, this is not routinely available in clinical practice.^131^

### 5.9 Megaesophagus

Megaesophagus describes the circumferential dilation of the esophagus (diffuse or segmental), with accompanying esophageal dysmotility.^232^ Megaesophagus (ME) is a well‐recognized cause of esophageal dysphagia in dogs and is considered an important risk factor for AARS.^207^, ^232^ Megaesophagus in dogs has been discussed extensively elsewhere but representative causes are presented in Table 2. Importantly, some dogs with presumed “idiopathic” ME have a functional obstruction of the lower esophageal sphincter (ie, LES‐AS).^52^ These dogs appear to respond to interventions targeting the LES including botulinum toxin injections, ballooning, and bougienage.^75^ Sildenafil has also shown promise in managing clinical signs in dogs with congenital idiopathic ME.^233^

**TABLE 2:** Representative causes of pharyngeal weakness in dogs.

Cranial nerve dysfunction
Hypoglossal nerve defects (CN 9)^30^
Viral
Rabies^31^
Pseudorabies^32^
Chronic upper airway obstruction
Brachycephalic obstructive upper airway syndrome^22^, ^33^
Epiglottic retroversion^34^
Tracheal collapse^35^
Toxicosis
Elapid snake envenomation^36^
Neuromuscular
Myasthenia gravis^37^, ^38^
Botulism^39^
Polyradiculoneuritis^37^
Myopathy
Muscular dystrophy^40^
Inflammatory myopathy^41^, ^42^
Polyneuropathies
Geriatric onset laryngeal paralysis polyneuropathy^43^
Traumatic (eg, penetrating stick, post operative)^44^, ^45^

**TABLE 3:** Representative causes of megaesophagus (ME) in dogs.

Focal
Vascular ring anomaly^50^
Esophageal stricture^50^
Esophageal masses^50^
Esophageal foreign body^51^
Generalized
Idiopathic
Congenital^50^
Acquired^50^
Lower esophageal sphincter achalasia‐like syndrome (LES‐AS)^52^
Endocrine
Hypoadrenocorticism^53^
± Hypothyroidism^54^
Neuromuscular
Myasthenia gravis^50^
Polyradiculoneuritis^50^
Tick paralysis^55^, ^56^
Muscular dystrophy^57^
Glycogen storage disease II^50^
Dysautonomia^50^
Immune mediated polymyositis^41^, ^42^
Generalized inflammatory myopathy^41^
Preneoplastic syndromes (eg, bronchogenic carcinoma, lymphoma, tonsillar carcinoma, myeloid leukemia)^50^
Laryngeal paralysis‐polyneuropathy (LP‐PNC and GOLPP)^43^, ^50^, ^58^
Dermatomyositis^59^
Demyelinating polyneuropathy^50^
Cervical vertebral instability with leukomalacia^60^
Brainstem lesions^50^
Systemic lupus erythematosus^60^
Toxicity
Lead^50^
Thalium^61^
Organophosphates^50^
Botulism^62^
Envenomation^55^
Tetanus^50^, ^63^
Acetylcholinesterase inhibitors^64^
Acrylamide^65^
Vincristine^66^
Infectious agents
Spirocerca lupi^67^
Neospora caninum^68^
Distemper^69^
Esophageal obstruction
Distal esophageal stricture^50^
Distal esophageal mass (ie, psuedoachalasia)^52^
Hiatal hernia (type 1 and type 2)^50^
Gastric dilatation and volvulus (GDV)^70^
Esophagitis^71^
Paraneoplastic syndromes^72^
Pituitary dwarfism^73^

Discrimination between ME and other causes of esophageal dilation (eg, aerophagia or sedation/anesthesia) is important to avoid inappropriate diagnosis and clinical decision making, including euthanasia.^232^ A VFSS is necessary to identify LES‐AS and discriminate ME from other forms of esophageal dilation.^52^

### 5.10 Esophagitis

Esophagitis refers to localized or diffuse inflammation of the esophageal mucosa. In most cases, this is the consequence of GER; however, focal injury because of esophageal foreign bodies (FB), medications (eg, doxycycline and clindamycin in cats) and eosinophilic esophagitis have also been reported.^74^, ^234^, ^235^, ^236^ Conditions that increase the frequency of reflux events such as gastric hyperacidity, sHH, gastric hypomotility, anesthesia, and upper airway obstruction should be considered predisposing factors for esophagitis.^13^, ^15^, ^172^, ^231^, ^237^, ^238^ Severe esophagitis is associated with the development of esophageal strictures, necrosis, and diverticulum formation as well as aspiration pneumonia/pneumonitis, pneumothorax, and tracheal compression.^234^ The decreased esophageal motility seen with esophagitis impairs the clearance of harmful gastric secretions introduced by reflux.^71^ Further, studies using animal models of esophagitis demonstrated diminished strength and frequency of the esophago‐laryngeal closure reflex (defined in Table 4), increasing risk of AARS.^239^ The diagnosis of esophagitis is multimodal, involving, but not limited to, VFSS, endoscopy, biopsies (eg, eosinophilic esophagitis), treatment trials, and ambulatory pH monitoring.^71^, ^74^

**TABLE 4:** A summary of airway protective mechanisms.

Airway protective mechanisms	
Normal swallowing	Coordinated conduction and clearance of food material and or secretions from the upper aerodigestive tract into the stomach^81^
Respiratory swallow coordination	
Gatekeeping	Centrally mediated coordination between respiration and swallowing to prevent both occurring simultaneously^82^, ^83^
Respiratory phase coordination	The phase of the respiratory cycle during which a swallow is triggered. This is variable across species^82^, ^83^, ^84^, ^85^, ^86^
Swallow apnea	Interruption of airflow corresponding to pharyngeal swallow^82^, ^83^

Basal mechanisms	
Lower esophageal sphincter (LES)	High pressure zone comprised of circumferential smooth muscle at the esophageal gastric junction (EGJ), external pressure from the diaphragm, rugal folds, and angle of entry of esophagus into the stomach^87^, ^88^ Barrier to gastric contents entering aerodigestive tract (ie, esophagus) In nonanesthetized dogs the basal pressure of the LES is approximately 30 mmHg though this varies with consciousness and body position (eg, reduced during sleep and anesthesia)^89^
Upper esophageal sphincter (UES)	Barrier between the upper airway and the esophagus. Tonic closure of the cricopharyngeus muscle prevents aerophagia during quiet breathing and orad movement of esophageal contents into the pharynx following pharyngeal swallow^90^
Residual volumes (pharynx and esophagus)	The finite volume of material the pharynx and esophagus have the capacity to contain before overflow into the airways and pharynx, respectively^83^, ^91^
Mucociliary apparatus	System comprised of ciliated epithelial cells and associated mucus layer that traps and clears aspirated material, as well as providing a biological and chemical barrier against injury^92^
Innate immunity	Antimicrobial defense mechanism involving leukocytes and epithelial cells lining the alveoli and conducting airways^93^

Response mechanisms	
Volume clearance	
Reflexive pharyngeal swallow (RPS)	Removes debris and residue from the pharynx and promotes clearance of refluxate from the esophagus by triggering primary esophageal peristalsis^94^, ^95^ Protective against spill‐over of oral contents during the oral/preparatory phase of swallowing, and orad movement of esophageal and gastric contents (eg, extraesophageal reflux (EER) and esophageal‐oropharyngeal reflux (EOR))^94^, ^96^, ^97^ RPS is not preceded by an oral/preparatory phase^94^ A higher threshold to trigger a RPS is considered a risk factor to aspiration in elderly people^96^, ^98^
Primary esophageal peristalsis	Esophageal clearance triggered by pharyngeal swallow.^99^ Conducts swallowed bolus toward the stomach. Retained material incompletely cleared by primary peristalsis may be cleared by subsequent secondary esophageal peristalsis
Secondary esophageal peristalsis	Vagally mediated reflex triggered by esophageal distension and not preceded by a pharyngeal swallow^100^, ^101^ Removes retained volume in the esophagus following primary peristalsis, or in response to refluxed material from the stomach^100^, ^101^
UES pressure	
Magnitude	The pressure of the UES is dependent on arousal, body position, and the degree, location, and rate of esophageal distention, as well as the distending material (gas vs liquid, acidic vs nonacidic)^90^, ^102^, ^103^
Esophago‐UES contractile reflex	Increased UES tone in response to slow, distal esophageal distention Rapid esophageal gas distention (ie, eructation) leads to UES relaxation. This reflex is nearly abolished during deep sleep^102^, ^104^
Pharyngo‐UES contractile reflex	Increased UES tone in response to pharyngeal stimulation unrelated to pharyngeal swallow. This is nearly always followed by RPS^104^, ^105^
Laryngo‐UES contractile reflex	Increase in UES tone in response to laryngeal stimulation^104^
Laryngeal closure	
Esophago‐laryngeal closure reflex	Laryngeal closure occurs in response to abrupt esophageal distention. Unlike the esophago‐UES reflex, abrupt esophageal distention causes laryngeal closure regardless of location^106^, ^107^
Pharyngeal‐laryngeal adduction reflex	Brief closure of the larynx in response to stimulation of the pharynx. This response is absent in patients that aspirate before pharyngeal swallow^108^, ^109^
Laryngeal adductor reflex	Adduction of the larynx in response to laryngeal stimulation^109^, ^110^, ^111^
Particulate clearing	
Cough	Airway protective mechanism triggered by chemical or mechanical stimulation of the pharynx, larynx, trachea, and bronchi^29^, ^112^, ^113^
Throat clear (TC)	Airway protective mechanism that differs from cough through the absence of an inhalation phase. Associated with stimulation of the upper respiratory tract (pharynx, larynx, cervical trachea)^112^, ^113^

**TABLE 5:** Mammalian penetration‐aspiration scale.^119^, ^153^

Classification	Score	Description
Normal	1	No material enters the supraglottic space during swallow
Penetration	2	Material enters the supraglottic space during swallow but is ejected before swallow completion
	3	A small volume of material enters the supraglottic space during swallow and remains after completion, or large amounts of material remain in the pharynx after swallow completion without entrance in to the supraglottic space
	4	A large amount of material enters the supraglottic space during a swallow and remains after swallow completion
Aspiration	5	Material falls caudal to the vocal folds but is ejected following cough
	6	Material falls caudal to the vocal folds and is not ejected despite coughing
	7	Material falls caudal to the vocal folds and no effort is made to eject the material (ie, occult aspiration)

*Note*: Scores ≥3 are considered pathologic.

### 5.11 Lower esophageal sphincter achalasia‐like syndrome (LES‐AS)

Lower esophageal sphincter achalasia‐like syndrome is a recently identified cause of megaesophagus and esophageal dysmotility in dogs caused by a failure of the LES to relax in response to a pharyngeal swallow.^52^ Importantly, LES‐AS responds to targeted intervention with botulinum toxin A injections and pneumatic dilation, as well as Heller myotomy with Dorr fundoplication.^75^ Though esophageal achalasia in people is diagnosed by manometry, currently the criterion standard for diagnosis of LES‐AS in dogs is VFSS.^52^

### 5.12 Reflux diseases: Gastro‐esophageal reflux disease (GERD), extraesophageal reflux disease (EERD), nasopharyngeal reflux disease (NPRD), esophago‐oropharyngeal reflux disease (EORD)

Gastroesophageal reflux disease and EERD, sometimes called laryngopharyngeal reflux disease, refer to clinical sequelae because of pathologic reflux with extension of gastric contents into the esophagus and the pharynx for GERD and EERD, respectively.^1^ Extension of EER or oropharyngeal contents into the nasopharynx is termed nasopharyngeal reflux (NPR; Figure 1).^240^ Esophageal contents refluxing back into the oropharynx without first entering the stomach is considered esophago‐oropharyngeal reflux (EOR). When NPR and EOR induce clinical signs and tissue injury, they are termed NPRD and EORD.

Like people, healthy dogs might have episodes of GER without apparent clinical consequence. Physiologic GER was detected in 41% of dogs by VFSS and in 100% by reflux scintigraphy.^10^, ^148^ As such, a diagnosis of abnormal reflux involves the volume, margination, frequency, and content of the refluxate rather than the absolute presence or absence of reflux events.^241^ Though sporadic EER events occur in asymptomatic people and dogs, these are largely considered abnormal and risk factors for AARS, with as few as 3 events being sufficient to cause detectable damage to upper airway structures.^10^, ^242^, ^243^

In human medicine, clinical signs of reflux are separated into “typical” and “atypical” forms. Typical reflux is associated with clinical signs of gastrointestinal disease (eg, heartburn and regurgitation).^244^ People with atypical reflux present with exclusively respiratory clinical signs.^245^ Gastroesophageal reflux in people has a prevalence of 50% in people with asthma, COPD, and chronic cough.^3^, ^5^, ^6^, ^7^ Extraesophageal reflux is implicated in chronic rhinosinusitis and chronic otitis media via eustachian tube.^246^ Despite the prevalence of GER in the aforementioned respiratory conditions, up to 45% of humans with confirmed GERD are asymptomatic for clinical signs of alimentary tract disease (ie, atypical GERD).^247^ Though less extensively characterized, there is an association between reflux and respiratory disease in dogs including lymphoplasmacytic rhinitis, laryngeal dysfunction, BOAS, idiopathic cough, and otitis media among others.^2^, ^14^, ^17^, ^18^, ^80^, ^172^, ^173^, ^248^, ^249^ EOR is an independent risk factor for abnormal laryngeal penetration and aspiration in dogs.^119^

Diagnosing reflux disease is a clinical challenge because of episodic reflux events, subtle or absent clinical signs of alimentary disease (ie, atypical reflux), and variable localization (ie, NPR vs GER). A VFSS is necessary to detect EOR and NPR and is sensitive enough to detect GER and EER.^2^, ^119^, ^120^ Small volume reflux and microaspiration events might require adjunctive measures including reflux scintigraphy.^10^ Ambulatory pH monitoring, though useful, might fail to detect nonacidic reflux events or those in dogs currently receiving proton pump inhibitors.^130^, ^250^ Treatment trials for GERD and EERD remain a mainstay in clinical practice. However, dogs undergoing appropriate treatment trials involving acid suppression, prokinetics, and lifestyle modification might take 6‐8 weeks to show a response.^23^, ^244^ The ACVIM guidelines on the use of gastrointestinal protectants in dogs and cats summarizes recommendations on the use of PPIs, to reach therapeutic targets of gastric pH from human medicine.^143^ Dosing should be selected to reach these recommended targets in an effort reduce possible placebo effect. Additionally, multimodal therapy including the use of prokinetics are recommended to address the frequency of reflux events.^143^

### 5.13 Sliding hiatal hernia

Sliding hiatal hernia (hiatal hernia type 1) is a contributor to both clinical signs of respiratory and alimentary disease in dogs. Displacement of the stomach through the esophageal hiatus reduces LES pressures predisposing dogs to pathologic reflux events, respiratory distress, and aspiration pneumonia.^2^, ^15^ Though mostly associated with brachycephalic breeds, recent studies have identified sHH in nonbrachycephalic dogs, and in dogs without clinical signs of alimentary disease.^15^ Brachycephalic dogs however, were younger upon diagnosis and more likely to present in respiratory distress than their nonbrachycephalic counterparts.^15^ Diagnosis of sHH has typically involved abdominal radiographs with or without abdominal compression, but this modality is poorly sensitive for detecting dynamic events such as reflux and accompanying esophageal dysmotility.^2^, ^13^, ^231^ VFSS is superior to thoracic radiographs for detecting sHH in dogs.^15^, ^231^

### 5.14 Structural diseases of the esophagus: strictures, persistent right aortic arch, diverticula

*Esophageal strictures*: Esophageal strictures are a consequence of esophagitis caused by reflux, esophageal foreign bodies, drugs, and anesthetic events among others.^71^, ^74^ Circumferential scar tissue forms a progressive barrier to esophageal clearance as well as increasing the residual volume of the esophagus proximal to the obstruction increasing risk of overload and aspiration.^94^

*Vascular ring anomalies*: Vascular ring anomalies such as persistent right aortic arch result in similar obstructive complications as strictures because of extraluminal compression of the esophagus.^251^

*Esophageal diverticulum*: An esophageal diverticulum, an outpouching of the esophageal wall, is an uncommon condition in dogs. These are largely classified by etiology. A pulsion diverticula is created in regions of increased intraluminal pressure. In people, pulsion diverticula are seen with cases of esophageal achalasia and esophageal dysmotility.^252^ Similar cases are reported in dogs.^52^ A traction diverticulum occurs when external forces are placed on the esophageal wall. In people, this is most associated with mediastinal inflammation.^252^ Most symptomatic patients present with signs of esophageal dysphagia; however, cases of occult (ie, only respiratory clinical signs or absent clinical signs) diverticula are reported in both people and dogs.^2^, ^52^, ^252^

Any impedance to esophageal clearance represents a risk factor for AARS.^23^, ^207^ A VFSS is helpful to identify a focal obstruction of the esophagus, such as those described above, as well as any accompanying AeroD (eg, esophageal dysmotility and reflux).^2^ Care should be taken not to mistake normal esophageal redundancy in brachycephalic dogs for an esophageal diverticula.^231^ Endoscopy is diagnostic and can be therapeutic for esophageal strictures.^253^ A CT with or without an angiogram might be required for identification and surgical planning for vascular ring anomalies and esophageal diverticula.^251^, ^252^ Endoscopy aided thoracoscopic correction of persistent right aortic arch has also been reported in dogs.^254^

## 6 CONCLUSIONS

Aerodigestive disorders are the result of discordant respiration and swallowing. Despite a high prevalence for such disorders in dogs presenting for clinical signs of respiratory disease, the link between abnormalities of breathing and swallowing is likely underrecognized in clinical veterinary practice. Advanced diagnostic and monitoring tools including videofluoroscopic swallow studies, scintigraphy, biomarker analysis, and objective respiratory biometrics are needed to improve recognition and treatment of these dogs. Further, as both dogs and people have aerodigestive disorders, using a One Health approach will increase the opportunity for bidirectional advancement in research and its clinical application.

## CONFLICT OF INTEREST DECLARATION

Authors declare no conflict of interest.

## OFF‐LABEL ANTIMICROBIAL DECLARATION

Authors declare no off‐label use of antimicrobials.

## INSTITUTIONAL ANIMAL CARE AND USE COMMITTEE (IACUC) OR OTHER APPROVAL DECLARATION

Authors declare no IACUC or other approval was needed.

## HUMAN ETHICS APPROVAL DECLARATION

Authors declare human ethics approval was not needed for this study.

## Supporting information


**VIDEO 1:** A video of a loop with a history of chronic cough and gagging is freely consuming kibble on videofluoroscopic swallow study (VFSS). Despite multiple appropriately timed swallow attempts, the kibble bolus is unable to pass through a hypertrophied upper esophageal sphincter (UES; cricopharyngeal achalasia). Multiple ineffectual swallow attempts lead to excessive pharyngeal accumulation of kibble and gagging.
